# Impact of Antibiotic Use in the Primary Treatment of Nasopharyngeal Carcinoma

**DOI:** 10.3390/cancers18132082

**Published:** 2026-06-26

**Authors:** Bojie Chen, Whitney T. Y. Ngan, Timothy Shun Man Chu, Cherrie W. K. Ng, Eddy W. Y. Wong, Eric H. L. Lau, Samuel C. C. Cheng, Catherine P. L. Chan, Andy H. K. Chan, David Johnson, Florence Mok, Daisy Lam, Kenneth C. W. Wong, Brigette Ma, Ka-Wai Kwok, Zigui Chen, Jason Y. K. Chan

**Affiliations:** 1 Department of Otorhinolaryngology, Head and Neck Surgery, The Chinese University of Hong Kong, Hong Kong SAR, China; jamiechen@cuhk.edu.hk (B.C.);; 2 Department of Clinical Oncology, The Chinese University of Hong Kong, Hong Kong SAR, China; 3 Department of Mechanical Engineering, The University of Hong Kong, Hong Kong SAR, China; 4 Depart of Mechanical and Automation Engineering, The Chinese University of Hong Kong, Hong Kong SAR, China; 5 Department of Microbiology, The Chinese University of Hong Kong, Hong Kong SAR, China

**Keywords:** nasopharyngeal carcinoma, antibiotics, chemoradiotherapy, survival, head and neck cancer

## Abstract

Background: Antibiotics are commonly prescribed to patients with nasopharyngeal carcinoma (NPC) during chemoradiotherapy; however, peri-treatment antibiotic use may adversely affect patients’ outcomes. Methods: A retrospective cohort study was conducted. The association between antibiotic use and patients’ survival time was analyzed using Kaplan–Meier and Cox proportional hazards regression models. Results: Among 455 NPC patients, 42.0% received antibiotics around primary treatment. Patients who had an advanced tumor stage (*p* = 0.019) or had received neoadjuvant chemotherapy (*p* = 0.008) or concurrent chemoradiotherapy (*p* = 0.002) were more likely to be prescribed antibiotics. Univariate analysis showed that antibiotic use around primary treatment was associated with worse disease-specific survival (DSS) at both 5 years (*p* = 0.043) and 10 years (*p* = 0.019). Subgroup analysis showed that 5-year and 10-year DSS were significantly shortened in patients receiving RT only and Abx within 2 w or 1 w around RT (5-year: 2 w *p* = 0.001, 1 w *p* < 0.001; 10-year 2 w *p* < 0.001, 1 w *p* = 0.005). Conclusions: In NPC, antibiotic use around primary treatment was associated with poorer disease-specific survival. Further prospective studies are warranted to clarify the causality and underlying mechanisms.

## Simple Summary

This study demonstrates that antibiotic exposure during primary chemoradiotherapy in patients with nasopharyngeal carcinoma is significantly associated with a worse tumor-specific survival. These negative effects are primarily driven by antibiotic-induced microbial dysbiosis, which subsequently disrupts host immune responses and impairs the therapeutic efficacy of chemoradiotherapy. Given the widespread use of antibiotics in oncological care to manage infections, these results highlight a critical need for clinicians to balance infection control against potential risks. More clinical research and further considerations are urgently required to refine precise antibiotic treatment strategies. Further focus could be placed on optimizing the timing of antibiotic administration to minimize gut microbiota disruption, ultimately safeguarding the efficacy of standard cancer therapies and improving patient prognoses.

## 1. Introduction

Nasopharyngeal carcinoma (NPC) is an epithelial carcinoma arising from the nasopharyngeal mucosa and presents unique epidemiological characteristics [1]. Despite a slightly decreased global incidence, NPC still ranks high among cancer morbidity and mortality in Southeast Asia [2]. In areas with a high incidence, non-keratinizing and undifferentiated carcinomas are the dominant subtypes, which are typically associated with the Epstein–Barr virus (EBV) [3]. Nowadays, intensity-modulated radiotherapy (IMRT) is greatly preferred in clinical practice over traditional two-dimensional (2D) or three-dimensional (3D) radiotherapy as it has less toxicity to important adjacent structures and provides better therapeutic outcomes [4]. Besides radiotherapy, it is well recognized that, in patients with regional lymph node metastases, concurrent chemoradiotherapy (CCRT) significantly improves prognosis [5]. For advanced stage III/IV NPC, induction or neoadjuvant chemotherapy (NC) is also recommended to be supplemented with CCRT for the purpose of improving patients’ survival.

An increasing number of studies imply that the gut microbiome may modulate the efficacy of cancer treatment, for instance, an attenuated response of rectal cancer to neoadjuvant chemoradiotherapy due to gut microbiota-mediated nucleotide synthesis has been reported [6,7]. In solid tumors, such as lung cancers, the negative impact of antibiotic exposure before immune checkpoint inhibitor treatment on the overall survival of older cancer patients has been observed in several medical centers [8]. Furthermore, antibiotic treatment is also reported to shorten overall survival (OS) and progression-free survival in chronic lymphocytic leukemia [9], relapsed lymphoma [9], and head and neck squamous cell carcinoma [10]. All these studies have aroused our interest in exploring the influence of antibiotic use on patient outcomes in the treatment of primary NPC.

Therefore, our study aimed to evaluate the effects of several clinical characteristics, especially the patterns of antibiotic use, such as the timing and frequency, on the prognosis of NPC patients, including patient survival and tumor recurrence.

## 2. Methods

### 2.1. Patients and Treatment

This retrospective study enrolled patients who were diagnosed with NPC and underwent treatment in our institution, a tertiary center in Hong Kong SAR, between January 2010 and December 2014. The study was approved by the Clinical Research Ethics Committee of the Joint CUHK-NTEC (CREC Ref. No. 2020.238). Patients were eligible for inclusion if they were: (1) primary nasopharyngeal carcinoma; (2) 18 years or older. Patients were excluded if they had: (1) prior history of malignancy; (2) previous history of irradiation to the head and neck region; (3) concurrent anti-inflammatory medications for concomitant diseases.

There were 455 patients identified. General characteristics of patients, including gender, age, history of smoking and drinking, and TNM staging (AJCC 7th edition), were obtained from electronic medical records. The information about their oncologic treatment, the scheme (induction/neoadjuvant chemotherapy, CCRT, RT alone), treatment start and end dates, and duration, were correspondingly collected. Referring to NCCN guidelines, for the mode of RT, intensity-modulated radiation therapy (IMRT) was most used, and the dose was usually 70 Gy for primary tumor and metastatic lymph nodes (2.12 Gy per time, q.d. and 5 times every week). Additionally, for concurrent chemoradiotherapy, cisplatin was prescribed at 40 mg/m^2^ per week or 100 mg/ m^2^ every 3 weeks; for neoadjuvant chemotherapy, the scheme of docetaxel and cisplatin and 5-fluorouracil or the one composed of gemcitabine and cisplatin was used. It was also recorded if patients received antibiotics (Abx), including the date and duration/courses of prescription, antibiotics type/class, the indication, and the mode of administration. Patients with antibiotic prescription within 4 weeks, 2 weeks or 1 week around their primary treatment (PTx) were further marked. For example, a patient received PTx from 1 June 2013 to 31 July 2013 and, if he took Abx treatment from 4 May to 28 August, he would be marked as ‘Abx around PTx’ and ‘Abx within 4 weeks around PTx’. If he got Abx treatment from 18 May to 14 August, he would be labeled as ‘Abx around PTx’ and ‘Abx within 2 weeks around PTx’. Only 5 records of Abx uses close to the date of PTx were included. The outcomes focused on were overall survival (OS), disease-specific survival (DSS) and recurrence-free survival (RFS).

The indications for Abx were based on common terminology criteria for adverse events (CTCAE) assessment on oral mucositis, infection of other sites (such as chest infection, ear infection), neutropenic fever, tuberculosis, and several individual cases. The classes of Abx were generally divided into β-lactam antibiotics and other types (including quinolone, gentamicin, isoniazid, rifampicin, etc.).

### 2.2. Outcomes

The following independent variables were examined to evaluate the association of antibiotics and NPC: (1) clinical factors: TNM staging of disease (AJCC 7th edition); (2) cancer risk factors: smoking history, alcohol consumption; (3) patient factors: sex, age; (4) treatment characteristics: chemotherapy, radiotherapy; (5) antibiotics: antibiotic use within 4 weeks around their primary radiotherapy ± chemotherapy including type, reason, duration, and date of prescription. We assumed that not all time points of antibiotics use would affect patient outcomes in relation to the primary treatment and consequently filtered out patients with antibiotics not within 4 weeks before or after their treatment.

Outcome measures included: recurrence-free survival—from time of diagnosis to time of recurrence locally, regionally and/or distantly. Disease-specific survival—from time of diagnosis to time of death from NPC. Overall survival—from time of diagnosis to time of death from all causes.

### 2.3. Statistical Analysis

Quantitative data were stated with mean ± standard deviation (SD) or median and interquartile range (IQR) according to their distribution. Qualitative data were shown with frequency and a chi-square test was performed for analysis of proportions across groups. OS, DSS and RFS were calculated from date of first diagnosis to tumor relapse or death, and the lost cases were censored at date of last follow-up. Uni- and multivariate survival analyses were carried out using Kaplan–Meier survival curves, log-rank tests or Breslow (generalized Wilcoxon) model tests, Cox proportional hazards and regression analysis respectively. A forward stepwise regression method was adapted in multivariable Cox regression analysis. All statistical tests were two-sided. A *p* value of less than 0.05 was considered statistically significant. Number of patients at risk has been added under the Kaplan–Meier survival plot to supplement related information. The data were analyzed with SPSS V27.0, R studio V2023.09.0 and GraphPad V8.0.

## 3. Results

A total of 455 NPC patients were included in this study. The mean age was 52.8 ± 11.4 years, and 344 patients (75.6%) were male. Most patients were diagnosed with stage III (n = 228, 50.1%) and stage IV (n = 109, 24.0%) NPC disease. Only one patient had stage IV disease with distant metastasis. Primary treatment involved CCRT (n = 379, 83.3%), which included CCRT only (n = 278, 61.1%) and NC followed by CCRT (NC + CCRT, n = 101, 22.2%). A small proportion received radiotherapy alone (n = 73, 16.0%). Patient characteristics, including history of tobacco and alcohol consumption, are summarized in Table 1. There were no significant differences in antibiotic use based on age (>50 years), gender, smoking status, or alcohol consumption. However, antibiotics use was significantly more frequent in patients with advanced tumor stage (AJCC 7th stage: *p* = 0.028, T stage: *p* = 0.003) and in those receiving concurrent chemotherapy (NC + CCRT: *p* = 0.008, CCRT only: *p* = 0.002). Conversely, it was less common in patients who only underwent radiotherapy alone (*p* < 0.001).

Patient outcomes are summarized in Table 2. The first death of a patient was noted on 21 November 2010, about 5 months after primary treatment, and the last follow-up of a surviving patient was recorded as 4 August 2021. The median follow-up was 87.5 months (IQR 65.0–107.6). During this period, 140 patients (30.7%) died, with 19.8% of deaths (n = 90) attributed specifically to NPC. Recurrence was observed in 106 patients (23.3%), including 64 (60.4%) developing distant metastases, 21 (19.8%) experiencing local recurrences, and 18 (17.0%) having regional recurrences. Five-year and ten-year OS rates were 79.8% and 69.9 respectively, and DSS rates were 85.5% and 80.4%. The median RFS was 85.6 months (IQR 41.8–106.5), with 5-year and 10-year RFS rates of 86.6% and 77.6%, respectively.

All antibiotic prescriptions in this study were administered for therapeutic purposes. Five records of 184 patients who accepted Abx around their PTx were included in this study. Among these patients, 85 patients (46.2%) received multiple courses of antibiotic (Abx), and 42 patients (22.8%) were treated with antibiotics from different classes. The distribution of antibiotic types include β-lactam only (n = 99, 53.8%), other classes (n = 47, 25.5%), and antibiotic combinations (n = 35, 19.0%). Since the number of antibiotic courses (from 1 to 5) varied per patient, a total of 322 valid antibiotic use records were analyzed and presented as person-time data. Among these, β-lactam antibiotics were the most frequently prescribed (n = 226, 70.2%), with penicillin-based medicine accounting for 192 uses (59.6%), followed by fluoroquinolone (n = 76, 23.6%) and cephalosporin (n = 34, 10.6%). The average duration of each antibiotic use was 8.12 ± 4.94 days. The three most frequently used antibiotics were Augmentin (n = 114; 109 oral, 5 intravenous), levofloxacin (n = 59; 58 oral, 1 intravenous), and piperacillin + tazobactam (n = 28; 20 oral, 8 intravenous). As shown in Table 3, patients who received antibiotics around PTx had a significantly lower DSS compared to those who did not receive Abx (5-year DSS: *p* = 0.043; 10-year DSS: *p* = 0.019). The negative impact on DSS was more pronounced in patients who received antibiotics within 2 weeks of PTx (5-year DSS, *p* = 0.044; 10-year DSS: *p* = 0.037). However, antibiotic use was not associated with a significantly increased risk of tumor recurrence (RFS: *p* = 0.350) or death of any cause (OS: *p* = 0.083). The most common clinical indication for antibiotics use was mucositis, a frequent adverse effect of chemoradiotherapy. Meanwhile, other antibiotics, such as clarithromycin, vancomycin, rifampicin, and isoniazid, were prescribed for treatment of infectious conditions including neutropenic fever and tuberculosis.

**Figure 1:**
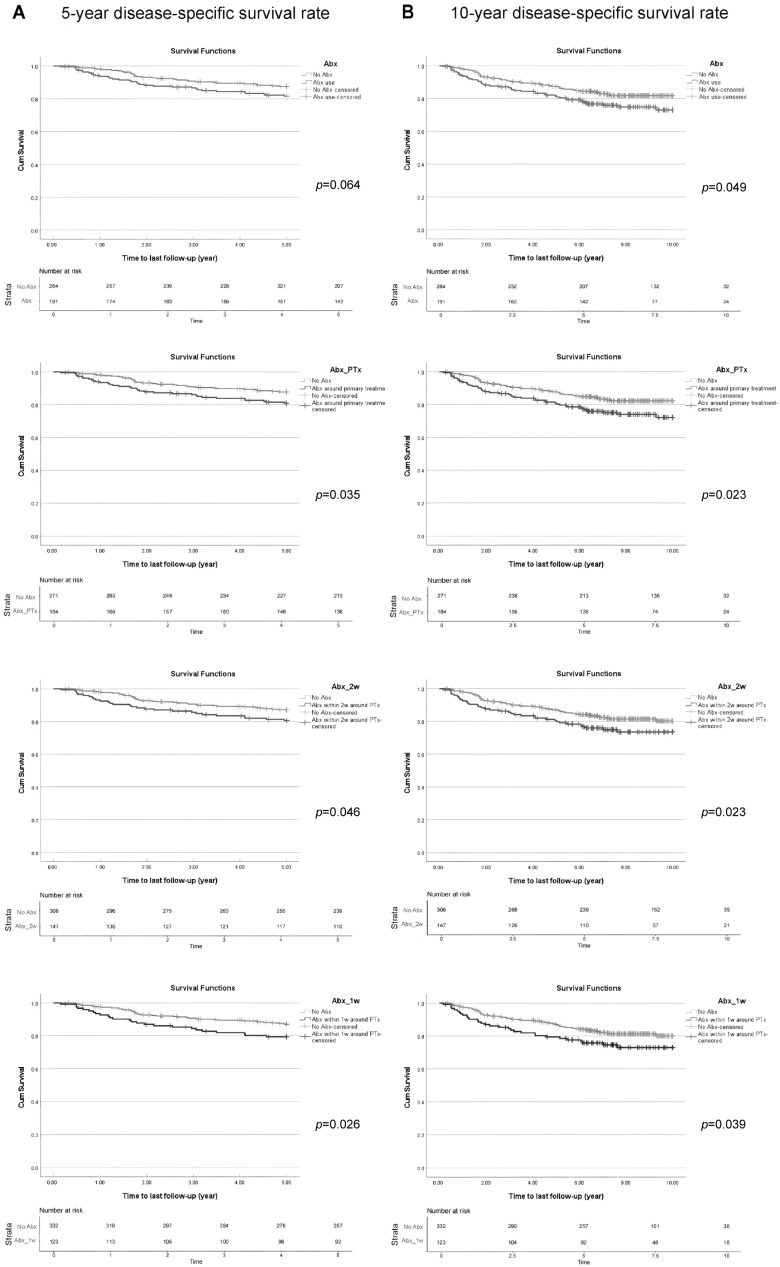
Survival curves for 5-year and 10-year DSS of NPC patients with and without antibiotic prescribed around their primary treatment. (**A**) The 5-year DSS rates of NPC patients affected by their antibiotic prescription, Abx around primary treatment, Abx use within 2 weeks or 1 week around PTx; (**B**) 10-year DSS rate changes.

The negative impacts of antibiotic use on 5-year and 10-year DSS rates of NPC patients is illustrated in Kaplan–Meier curves (Figure 1). Univariate Cox regression analysis confirmed that antibiotics use around PTx was significantly associated with a worse DSS at both 5 year and 10 years (5-year hazard ratio, HR, 1.644, 95% confidence interval, 95% CI [1.015, 2.665], *p* = 0.043; 10-year HR = 1.640 [1.085, 2.480], *p* = 0.019) (Table 3). Furthermore, when stratified by time window, antibiotic use within 2 weeks of PTx was also associated with poorer DSS outcomes (5-year *p* = 0.044, 10-year *p* = 0.037). Similarly, patients who received antibiotics within 1 week of PTx had poorer 5-year DSS (5-year *p* = 0.026; 10-year 1 week *p* = 0.060). Indicators of poor prognosis included oral antibiotic administration (5-year HR = 1.731 [1.069, 2.805], *p* = 0.026; 10-year HR = 1.647 [1.087, 2.497], *p* = 0.019) and non-β-lactam antibiotics (5-year HR = 2.530 [1.331, 4.807], *p* = 0.005; 10-year HR = 2.215 [1.236, 3.969], *p* = 0.008).

Univariate analysis also revealed several factors significantly associated with poorer DSS in NPC patients (Table 3), including current smokers (5-year DSS *p* = 0.012; 10-year DSS *p* = 0.024), advanced stage (5-year DSS *p* < 0.001; 10-year *p* < 0.001), lymph node metastasis (LNM) (5-year *p* = 0.026; 10-year *p* = 0.004), neoadjuvant chemotherapy (NC) (5-year *p* < 0.001; 10-year: *p* < 0.001), and antibiotic use around the time of PTx (5-year *p* = 0.043; 10-year *p* = 0.019). These includes current smoker (HR = 2.022 [1.165, 3.511], *p* = 0.012) and antibiotic use around PTx (HR = 1.644 [1.015, 2.665], *p* = 0.043), particularly when antibiotics were administered within 2 weeks of PTx (HR = 35.996 [3.999, 323.979], *p* = 0.001) or 1 week (HR = 42.932 [4.759, 387.309], *p* < 0.001). Other negative predictors included the use of non-β-lactam antibiotics (other classes of antibiotics) (HR = 2.530 [1.331, 4.807], *p* = 0.005) and oral antibiotic administration (HR = 1.731 [1.1069, 2.805], *p* = 0.026). For 10-year DSS, significant risk factors included older age (HR = 1.101 [1.038, 1.168], *p* = 0.001), any antibiotic use (HR = 4.282 [1.237, 14.828], *p* = 0.022), and antibiotic use around PTx (HR = 5.411 [1.559, 18.782], *p* = 0.008) especially within 2 weeks (HR = 12.447 [3.521, 44.006], *p* < 0.001) or 1 week (HR = 7.959 [1.861, 34.043], *p* = 0.005). Other high-risk indicators included receiving multiple courses of antibiotics (HR = 12.366 [3.257, 46.951], *p* < 0.001), use of combined classes of antibiotics (HR = 38.094 [6.17, 235.187], *p* < 0.001) and oral antibiotic administration (HR = 5.411 [1.559, 18.782], *p* = 0.008).

**Figure 2:**
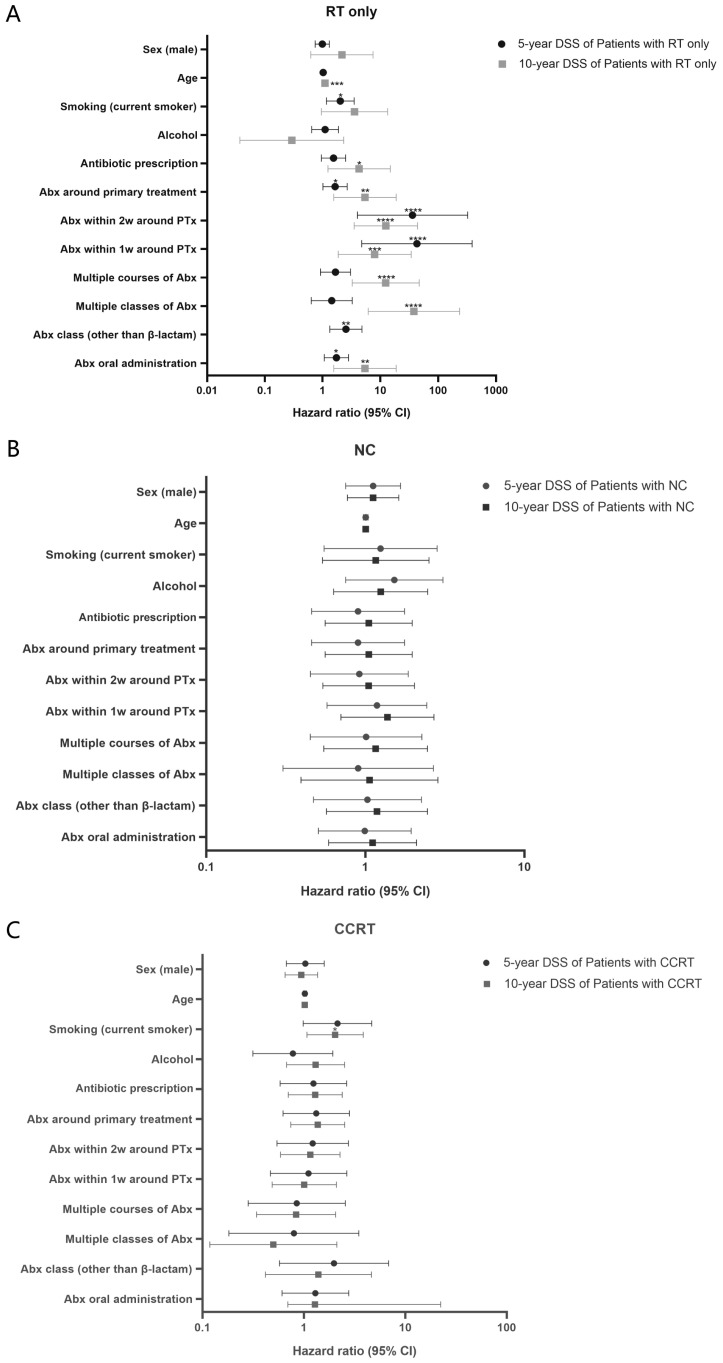
The influencing factors of 5-year and 10-year DSS of NPC patients with different primary treatment. (**A**) Patients who received RT only; (**B**) patients receiving NC; (**C**) patients receiving CCRT. Statistical significance is indicated as * *p* < 0.05, ** *p* < 0.01, *** *p* < 0.001, and **** *p* < 0.0001.

In Figure 2, subgroup analysis stratified by treatment strategy is shown in terms of 5-year and 10-year DSS. In patients receiving RT only, who were more likely to have a tumor at an early stage, several factors were significantly associated with worse 5-year DSS (Figure 2A). However, these negative effects were not found in patients receiving NC or CCRT (Figure 2B,C).

The multivariate model included age, smoking status, tumor stage and antibiotic use around PTx. For clinical and statistical considerations, lymph node metastasis (LNM) was excluded as it was already accounted for in the assessment of tumor clinical stage. Likewise, NC was also excluded since treatment choices were determined based on tumor clinical stage in clinical practice. The exclusion of NC to avoid multicollinearity was justified by a chi-square test that confirmed a strong correlation between advanced tumor stage and receipt of NC (*p* < 0.001). As shown in Table 4, advanced tumor clinical stage emerged as the most significant predictor of poor DSS rates (5-year DSS: HR = 5.759 [2.089, 15.875], *p* < 0.001; 10-year DSS: HR = 3.008 [1.555, 5.819], *p* = 0.001). Although antibiotic use around PTx did not reach statistical significance, its elevated hazard ratio remained noteworthy, particularly antibiotic use within 1 week of PTx for 5-year DSS (*p* = 0.087) and antibiotic use around PTx for 10-year DSS (*p* = 0.062). To further explore the prognostic impact of antibiotics, patients were stratified into subgroups based on survival time: short-term survivors (<5 years) and long-term survivors (≥5 years). Although the long-term survival group did not show any significant association of variables with poor prognosis, antibiotic use around PTx in the short-term survival group was a strong independent predictor of poor outcomes in the multivariate model (HR = 1.793 [1.099, 2.924], *p* = 0.019). In contrast, age (*p* = 0.056) and tumor stage (*p* = 0.115) did not show significant effects in this subgroup.

## 4. Discussion

This study focused on the effects of antibiotics use on treatment outcomes in patients with primary nasopharyngeal carcinoma. Our findings suggest that antibiotic use around the period of primary treatment may be associated with poorer disease-specific survival in NPC patients. The timing, the choices and combinations of drugs, and even the administration of Abx would lead to different patient outcomes, especially in patients with an earlier tumor stage. Regardless, antibiotics use within 4 weeks was associated with a worse disease-specific survival in both the short and long terms in NPC patients. However, this association may be influenced by underlying clinical conditions or tumor burden that necessitate antibiotic administration. The underlying cause may not only be attributed to the direct impact of antibiotics themselves on cancer treatment, but rather primarily to clinical indications for antibiotic use that potentially lead to rapid tumor deterioration and disease progression.

In our study, the subgroup analysis in the RT-only group and NC/CCRT group revealed that the negative impact of Abx around PTx on 5-year and 10-year DSS was exclusively significant in the RT-only patient cohort. Patients receiving radiation therapy alone were predominantly at an early clinical stage, and they were more sensitive to antibiotic-driven microbiota disruption. As for NPC patients with advanced-stage disease, the primary treatment strategy centers on radiotherapy combined with chemotherapy (including concurrent chemotherapy and neoadjuvant or adjuvant chemotherapy). Intensive RT with or without CT results in mucositis, increasing the risk of bacterial infection and the possibility of treatment suspension as well [11]. Immunosuppression caused by chemotherapy can also increase the risk of infection [12]. Many classes of chemotherapy drugs induce acute myelosuppression [13], impairing immune responses of patients and increasing the risks of infection [14]. Moreover, neutropenia, one of the most common hematological toxicities of chemotherapy, has a high morbidity [14]. Therefore, at this point, the antibiotic is then prescribed for its antimicrobial benefits in managing severe complications, such as mucositis and neutropenia, which are likely to counterbalance the harms of microbial disturbance. In our cohort, Abx were more commonly prescribed in patients receiving CCRT or NC, since a large proportion of these patients suffered neutropenic fever, oral mucositis and infections at other sites, similar to other studies [15]. Conclusively, antibiotic prescriptions require careful consideration to balance benefits and harms. For NPC patients at early stages or just receiving radiotherapy, the Abx should be carefully taken since they may contribute to microbial disturbance and bring negative effects to primary treatment; for advanced tumor cases, the Abx administration could be prescribed strictly according to the indications. In line with our findings, Weng et al. reported that, within an NPC cohort sustaining severe mucositis, patients receiving antibiotic therapy sustained significantly worse OS and DFS. Their multivariate Cox regression analysis identified tumor size, nodal status, therapeutic strategies, and antibiotic administration as significant independent predictors of survival outcomes. These peer-published data validate that the detrimental impact of antibiotic exposure remains robust even after rigorously adjusting for tumor burden [16].

Chiang et al. conducted a large-scale cohort study to monitor the survival of 7893 Hong Kong NPC survivors with a 20-year follow-up [17]. They reported that up to 59.9% of long-term deaths were attributed to non-cancer complications, primarily recurrent aspiration pneumonia driven by radiation-induced dysphagia and neck fibrosis [17]. The treatment of late-onset aspiration pneumonia routinely involves antibiotics, which would inevitably introduce overwhelming confounding by indication and mask any potential impact of Abx on definitive treatment efficacy. Consequently, we restricted the Abx exposure timeline period to filter out possible noise and ensure a pure biological read. This methodological strategy was also adapted by other studies. Notably, Fang et al. investigated the impact of antibiotics on the efficacy of first-line immunotherapy in recurrent or metastatic NPC, and they strictly defined the exposure windows as 30 days before and after the treatment [18]. In this study, the definition of ‘Abx use around PTx’ was proposed to prevent overestimating the potential effects brought by antibiotics on patient’s survival. Similarly, their findings demonstrated that Abx use in this window led to a significantly shorter overall survival. The restriction of the observation window helps to filter out irrelevant longitudinal noise, whereas this setting lacks a strict order between Abx and primary treatment, which may limit our ability to deduce a definitive cause-and-effect relationship. The oral microbiome may be altered by chemoradiotherapy and can potentially exacerbate mucositis. It is widely recognized that radiotherapy causes mucosal damage by release of reactive oxygen species (ROS), activation of transcription factors, including NF-ĸB, and upregulation of proinflammatory cytokines [19]. Furthermore, CCRT induces apoptosis of basal epithelial cells, which aggravates injury to the oral epithelial barrier and eventually promotes the development of mucositis in virtually every patient receiving combination therapy [20]. Current studies hold controversial views about whether the dynamically altered structure of oral microbiota during RT could be related to the occurrence of severe RT-induced mucositis [21]. Zhu et al. reported that lower bacterial alpha diversity and a higher abundance of *Actinobacillus*, a genus of Gram-negative bacteria, predicted aggregation of mucositis at a high predictive accuracy [22], similarly to the study of Reyes-Gibby et al. [23]. However, Vesty et al. found that salivary microbiota, which were dominantly composed of *Streptococcus*, *Prevotella*, *Fusobacterium* and *Granulicatella*, remained stable during treatment [24]. Hou et al. also supported this where they observed any significant fluctuation in overall richness or evenness of the oral microbiome during RT, whereas they noticed that bacterial proportions varied greatly, for instance, a decrease in *Prevotella* and *Fusobacterium* but an increase in *Granulicatella* [25]. Several clinical trials and research provided some other supporting evidence. Jiang et al. conducted a randomized controlled trial (NCT03112837) on patients receiving probiotic combination versus placebo during CCRT and the incidence of severe oral mucositis (grade 3 or higher), the number of immune cells, and intestinal microbial diversity of NPC [26]. They found that patients taking probiotics during CCRT were more likely to have low-grade oral mucositis and high levels of CD3+ T cells, CD4+ T cells and CD8+ T cells compared to the placebo group, which was thought to dampen the inflammatory response and restore gut microbiota to normalcy [26].

Host immunity is linked with the gut microbiome and maintains hemostasis in physiological conditions [27]. In return, gut microbes help to establish local immunity by building up colonic mucosal barriers [28]. Numerous studies have proved that gut flora makes a difference to therapeutic effects of chemoradiotherapy in anticancer treatment via numerous pathways: innate lymphoid cell and CD4+ regulatory T cell differentiation [29,30], interleukin expression [27,31,32,33,34], myeloid differentiation primary response 88 [28,32], etc. Butyrate, the most common short-chain fatty acid produced by gut microbiota, inhibited radiation-induced antitumor immune response by suppressing interferon expression in dendritic cells, CD8+ T cell differentiation and activation of antigen-presenting cells [35,36,37]. Cyclophosphamide (CTX), a widely used chemotherapy drug, causes effects by inducing immunological tumor cell death, meanwhile it promotes inward translocation of Gram-positive bacteria. As a result, the ratio of intratumoral CD8+ T cells to Treg cells is elevated, and both innate and adaptive immune responses are stimulated, characterized by activated pathogenic T helper (TH) 17 cells and memory TH1 cells [37,38]. If antibiotics covering specific flora are used in concurrence with CTX, the antitumor effect is weakened [38]. Gehrke et al. reported the radio-sensitizing property of salinomycin, together with its coverage against Gram-positive bacteria, which amplified the efficiency of RT in head and neck squamous cell carcinoma cells in vitro [39]. A similar effect was discovered in the study of Aydemir et al. [40], where they assayed concurrent effects of levofloxacin with cisplatin on oral squamous cell carcinoma cells, which suggested that levofloxacin could augment the effectivity of cisplatin and reduce its toxicity as well. The negative effects of antibiotics on patients’ prognoses and the efficacy of chemoradiotherapy are also observed in other malignancies, such as colorectal carcinoma [41], non-small-cell lung cancer [42], and ovarian cancer [43].

Our work aimed to specify the effects of concurrent antibiotic administration on NPC patients’ primary treatment and thus their survival outcomes and tumor recurrence. Several limitations of this study warrant mention. First, our study was retrospective, which is associated with selection bias and other potential offsets. In addition, in our cohort, up to 75% of patients had advanced stage diseases. The bias caused by tumor stage was emphasized after grouping. It also means that the sample size of patients with early-stage tumors was insufficient for further subgroup analysis due to a low number of deceased patients. While most of these patients required only radiotherapy and showed good prognosis, our study showed that antibiotics administration poses harmful effects on the survival outcomes of this population as well. Therefore, the negative impact of antibiotics on the outcomes of patients with advanced NPC could be confounded by tumoral factors and the corresponding therapeutic strategies. As for the patients with early tumor stage and receiving radiotherapy only, the sample size of this population was too small (73 patients in total and 17 patients receiving Abx), which was represented by statistical instability with extremely high HR values and a large 95% CI gap. This sparse data bias may overestimate the adverse impact of antibiotic use around primary treatment on the prognosis. In addition, clinical samples were not collected for this study. There was no way to further clarify the effects of antibiotic administration on the microbiome and host immune system, which could be an important focus in future studies. Furthermore, due to limited information extracted from electronic medical records, the exact indication for each patient with Abx use was lacking. For this reason, there was no way for us to know whether the planned cancer therapies for these patients were altered, such as reducing the dose. It is still inconclusive that the strong correlation between Abx use and poor survival within 5 years was due to the negative effects of the antibiotic itself on cancer treatment or the indications for the Abx, such as chest infection, sepsis, etc. We need more prospective clinical studies as well as more detailed medical records to facilitate further investigations. Another limitation is that our cohort was evaluated using the AJCC 7th edition, which differs from the current 8th edition. The major differences between these two versions include prioritization of induction chemotherapy and specific staging criteria, particularly pterygoid muscle involvement. Due to limited raw records of our cohort information, such as exact tumor sites or lymph node invasion, a retrospective re-staging and analysis according to the 8th edition were not feasible. Additionally, EBV infection has been widely regarded as a nearly indispensable factor in NPC pathogenesis. However, we have not detected the exact EBV titer in these patients because EBV infection is endemic in Hong Kong. Therefore, we could not explore any clue indicating the effect of antibiotics on EBV infection and related NPC tumor characteristics. Moreover, since strong correlations were explored in advanced tumor stage and many other indicators, such as NC treatment and Abx use as well, the result of multivariate Cox regression analysis would be influenced by their mutual effects. The current evidence is insufficient to define antibiotic use as an independent risk factor for poor prognosis. Establishing such a conclusion requires larger, more rigorous prospective cohorts and bench-side mechanistic proofs.

Under current AJCC 8th edition guidelines, the utilization of NC with RT or CCRT regimens for NPC treatment inevitably leads to a higher incidence of severe complications, such as acute mucositis. It is a critical issue to optimize rational antibiotic administrations. Our study stresses that antibiotic use during NPC primary treatment could be viewed as a clinical indicator. This marker can remind clinicians to avoid unnecessary Abx use in early NPC patients, decide appropriate Abx use outside of NPC primary treatment, keep alert to the adverse events of intense cancer therapy, strictly prescribe the Abx according to the indications, and positively monitor tumor recurrence in patients with advanced tumor and Abx administration.

## 5. Conclusions

Antibiotic administration in the clinical setting possesses both benefits and harms and should be done under careful consideration. We identified its negative effects on tumor-specific survival in nasopharyngeal carcinoma, especially when it is given around the period of primary treatment. By disrupting microbiomes and thereby modulating the host immune system, antibiotics could potentially impact the efficacy of chemoradiotherapy in cancer treatment. Further exploration of antibiotic-related microbial dysbiosis may identify potential novel therapeutic strategies that improve survival outcomes in cancer patients.

## Figures and Tables

**Table 1:** The characteristics of NPC patients receiving primary treatment with or without antibiotics use.

Characteristics	Total Number (n = 455, %)	Antibiotics		
		Yes (n = 191, 42.0%)	No (n = 264, 58.0%)	*p* Value
Sex				0.595
Female	111 (24.4%)	49 (25.7%)	62 (23.5%)	-
Male	344 (75.6%)	142 (74.3%)	202 (76.5%)	
Age	52.8 ± 11.4	52.5 ± 11.4	53.0 ± 11.4	0.612
Smoking status (Current smoker as reference)				0.209
Current smoker	109 (24.0%)	39 (20.4%)	70 (26.5%)	-
Ex-smoker	96 (21.1%)	46 (24.1%)	50 (18.9%)	0.079
Non-smoker	250 (54.9%)	106 (55.5%)	144 (54.5%)	0.240
Alcohol usage				0.467
Drinker	120 (26.4%)	47 (24.6%)	73 (27.7%)	-
Non-drinker	335 (73.6%)	144 (75.4%)	191 (72.3%)	
Staging (AJCC 7th) ^a^ (Stage IV as reference)				0.028 *
I	31 (6.8%)	7 (3.7%)	24 (9.1%)	0.008 *
II	85 (18.7%)	31 (16.4%)	54 (20.5%)	0.053
III	228 (50.1%)	96 (50.8%)	132 (50%)	0.150
IV	109 (24.0%)	55 (29.1%)	54 (20.5%)	-
T stage (AJCC 7th) ^a^ (T4 stage as reference)				0.003 *
T1	163 (36.0%)	50 (26.5%)	113 (42.8%)	0.003 *
T2	48 (10.6%)	25 (13.2%)	23 (8.7%)	0.944
T3	172 (38.0%)	78 (41.3%)	94 (35.6%)	0.391
T4	70 (15.5%)	36 (19.0%)	34 (12.9%)	-
N stage (AJCC 7th) ^a^ (N0 stage as reference)				0.857
N0	67 (14.8%)	27 (14.1%)	40 (15.2%)	-
N1	154 (34.0%)	64 (33.5%)	90 (34.1%)	0.861
N2	179 (39.5%)	73 (38.2%)	106 (40.2%)	0.945
N3	53 (11.7%)	25 (13.1%)	28 (10.6%)	0.451
Treatment received ^b^ (NC + CCRT as reference)				<0.001 *
RT only	73 (16.0%)	17 (8.9%)	56 (21.2%)	<0.001 *
CCRT only	278 (61.1%)	119 (62.3%)	159 (60.2%)	0.066
NC + CCRT	101 (22.2%)	54 (28.3%)	47 (17.8%)	-

^a^ Tumor stages of 2 cases were lost; ^b^ 3 cases received NC + RT; * *p* value < 0.05 shows statistical significance.

**Table 2:** The outcomes and treatment scheme of NPC patients with antibiotics use.

	Total Number (n = 455) (%)/(Median, IQR)	Antibiotics		
**Antibiotic prescription**		**Yes (n = 191)**	**No (n = 264)**	***p* Value**
Follow-up (month)	87.5 (65.0–107.6)	84.3 (55.4–107.6)	90.0 (71.8–107.9)	0.063
Outcomes				
Deceased	140 (30.8%)	65 (34.0%)	75 (39.3%)	0.200
Survived	315 (69.2%)	126 (66.0%)	189 (60.7%)	
Died of disease	90 (19.8%)	46 (24.1%)	44 (16.7%)	0.179
Other causes	50 (11.0%)	19 (9.9%)	31 (11.7%)	0.138
Survived with recurrence	26 (5.7%)	8 (4.2%)	18 (6.8%)	0.071
Disease-free survived	289 (63.5%)	118 (61.8%)	171 (64.8%)	0.087
Recurrence				0.268
Recurrence-free survived	289 (63.5%)	118 (61.8%)	171 (64.8%)	0.513
Survived with recurrence	26 (5.7%)	8 (4.2%)	18 (6.8%)	0.319
Deceased	140 (30.8%)	65 (34.0%)	75 (28.4%)	0.272
**Antibiotic prescription around primary treatment**		**Yes (n = 184)**	**No (n = 271)**	***p* value**
Follow-up (month)	**-**	84.4 (55.0–107.3)	89.9 (72.0–108.0)	0.067
Outcomes				
Deceased	140 (30.8%)	65 (35.3%)	75 (27.7%)	0.083
Survived	315 (69.2%)	119 (64.7%)	196 (72.3%)	
Died of disease	90 (19.8%)	46 (25.0%)	44 (16.2%)	0.083
Other causes	50 (11.0%)	19 (10.3%)	31 (11.4%)	0.138
Recurrence				0.111
Recurrence-free survived	289 (63.5%)	112 (60.9%)	177 (65.3%)	0.334
Survived with recurrence	26 (5.7%)	7 (3.8%)	19 (7.0%)	0.237
Deceased	140 (30.8%)	65 (35.3%)	75 (27.7%)	0.131
**Abx scheme**				
Multiple courses	-	85 (46.2%)	-	-
Multiple classes	-	42 (22.8%)	-	-
Classes				
β-lactam	-	99 (53.8%)	-	-
Cocktail/Multiple	-	35 (19.0%)	-	-
Other	-	47 (25.5%)	-	-
**Abx Timing (around primary treatment)**				
Within 4 weeks	-	184 (100.0%)	-	-
Within 2 weeks	-	147 (79.9%)	-	-
Within 1 week	-	123 (66.8%)	-	-

**Table 3:** Univariate Cox regression analysis of factors affecting DSS of NPC patients.

	5-Year DSS		10-Year DSS	
	*p* Value	HR (95% CI)	*p* Value	HR (95% CI)
Gender	0.930	0.987 (0.745, 1.308)	0.815	1.029 (0.812, 1.304)
Age	0.057	1.021 (0.999, 1.044)	0.018 *	1.023 (1.004, 1.042)
Smoking				
Non-smoker	-	-	-	-
Ever smoker	0.253	1.442 (0.770, 2.699)	0.499	1.205 (0.701, 2.073)
Current smoker	0.012 *	2.022 (1.165, 3.511)	0.024 *	1.727 (1.076, 2.772)
Alcohol	0.720	1.102 (0.647, 1.878)	0.541	1.154 (0.730, 1.824)
Stage				
Stage I	- ^a^	-	-	-
Stage II	-	-	0.269	3.206 (0.406, 25.307)
Stage III	-	-	0.100	5.308 (0.728, 38.688)
Stage IV	-	-	0.007 *	15.227 (2.095, 110.676)
Stage group				
Early stage (I + II)	-	-		
Advanced stage (III + IV)	<0.001 *	5.784 (2.103, 15.909)	<0.001 *	3.136 (1.625, 6.054)
LNM	0.026 *	26.298 (1.479, 467.478)	0.004 *	17.546 (2.440, 125.948)
NC	<0.001 *	4.164 (2.567, 6.754)	<0.001 *	3.220 (2.119, 4.894)
CCRT	0.183	1.702 (0.778, 3.727)	0.385	1.309 (0.713, 2.404)
Antibiotic use				
No Abx	-	-	-	-
With Abx	0.077	1.546 (0.954, 2.505)	0.040 *	1.541 (1.020, 2.331)
With Abx around primary treatment	0.043 *	1.644 (1.015, 2.665)	0.019 *	1.640 (1.085, 2.480)
Abx use within 2 weeks around PTx	0.044 *	1.685 (1.015, 2.799)	0.037 *	1.600 (1.030, 2.486)
Abx use within 1 week around PTx	0.026 *	1.811 (1.073, 3.055)	0.060	1.562 (0.982, 2.484)
β-lactam use	0.414	1.29 (0.701, 2.374)	0.231	1.364 (0.821, 2.268)
Other Abx use	0.005 *	2.53 (1.331, 4.807)	0.008 *	2.215 (1.236, 3.969)
Antibiotic oral administration	0.026 *	1.731 (1.069, 2.805)	0.019 *	1.647 (1.087, 2.497)

^a^ None of the Stage I patients died of NPC within 5 years; * *p* value < 0.05 was considered statistically significant.

**Table 4:** Multivariate Cox regression analysis of factors affecting DSS of NPC patients.

	5-Year DSS		10-Year DSS	
	*p* Value	HR (95% CI)	*p* Value	HR (95% CI)
Age	0.142	-	0.204	-
Stage (advanced)	<0.001 *	5.759 (2.089, 15.875)	0.001 *	3.008 (1.555, 5.819)
Abx around primary treatment	-	-	0.062	1.488 (0.981, 2.257)
Abx within 1 w around PTx	0.087	1.547 (0.939, 2.547)	-	-

* *p* value < 0.05 was considered statistically significant.

## Data Availability

All data generated or analyzed during this study are included in this published article and Supplementary Files.

## Supplementary Materials

The following supporting information can be downloaded at: https://www.mdpi.com/article/10.3390/cancers18132082/s1, Table S1: Univariate Cox regression analysis of the factors affecting recurrence of NPC patients; Table S2: Univariate Cox regression analysis of the factors affecting overall survival status of NPC patients; Table S3: Treatment period and physical baseline of NPC patients with or without Abx.

## Abbreviations

The following abbreviations are used in this manuscript:

NPC	Nasopharyngeal carcinoma
Abx	Antibiotics
EBV	Epstein–Barr virus
IMRT	Intensity-modulated radiotherapy
2D	Two-dimensional
3D	Three-dimensional
RT	Radiotherapy
CCRT	Concurrent chemoradiotherapy
NC	Neoadjuvant chemotherapy
Gy	Gray
PTx	Primary treatment
OS	Overall survival
DSS	Disease-specific survival
RFS	Recurrence-free survival
AJCC	American Joint Committee on Cancer
CTCAE	Common terminology criteria for adverse events
SD	Standard deviation
IQR	Interquartile range
LNM	Lymph node metastasis
TH	T helper
CTX	Cyclophosphamide
