# Supplementary material for: Impact of Antibiotic Use in the Primary Treatment of Nasopharyngeal Carcinoma
Source: Cancers (Basel). 2026 Jun 26;18(13):2082. doi: 10.3390/cancers18132082 (PMC13359527; doi:10.3390/cancers18132082)
Supplement: Supplementary file 1 [file cancers-18-02082-s001.zip › Supplementary Table S1.pdf]

Supplementary Table S1 Univariate Cox regression analysis of the factors affecting recurrence of NPC patients.

	Overall RFS		5-year RFS		10-year RFS	
	P value	HR, 95% CI	P value	HR, 95% CI	P value	HR, 95% CI
Gender	0.732	1.078 (0.7, 1.661)	0.810	1.059 (0.665, 1.686)	0.720	1.082 (0.703, 1.666)
Age	0.901	0.999 (0.982, 1.016)	0.670	0.996 (0.978, 1.015)	0.918	0.999 (0.982, 1.017)
Smoking						
Ever smoker	0.794	1.071 (0.639, 1.794)	0.313	1.314 (0.773, 2.233)	0.782	1.076 (0.642, 1.802)
Current smoker	**0.020***	1.67 (1.084, 2.73)	**0.025***	1.713 (1.07, 2.744)	**0.019***	1.677 (1.088, 2.584)
Alcohol	0.716	1.082 (0.709, 1.651)	0.745	1.078 (0.685, 1.697)	0.704	1.085 (0.711, 1.656)
Stage (vs Stage I)						
Stage II	0.191	2.673 (0.611, 11.69)	0.545	1.606 (0.347, 7.431)	0.191	2.673 (0.611, 11.69)
Stage III	0.101	3.268 (0.793, 13.472)	0.151	2.834 (0.684, 11.737)	0.103	3.252 (0.789, 13.407)
Stage IV	**0.003***	8.278 (2.006, 34.163)	**0.005***	7.614 (1.842, 31.469)	**0.003***	8.278 (2.006, 34.162)
Stage (Early vs advanced)	**0.006***	2.084 (1.24, 3.502)	**<0.001***	2.899 (1.544, 5.444)	**0.006***	2.077 (1.236, 3.49)
LNM	**0.001***	6.744 (2.14, 21.259)	**0.003***	5.777 (1.828, 18.255)	**0.001***	6.847 (2.172, 21.582)
NC	**<0.001***	2.552 (1.722, 3.781)	**<0.001***	3.059 (2.022, 4.628)	**<0.001***	2.577 (1.739, 3.819)
CCT	0.068	1.787 (0.957, 3.336)	0.120	1.683 (0.873, 3.246)	0.073	1.77 (0.948, 3.304)
Abx	0.268	1.241 (0.847, 1.82)	0.205	1.304 (0.865, 1.963)	0.290	1.229 (0.839, 1.802)
Abx around primary Tx	0.223	1.269 (0.865, 1.862)	0.181	1.323 (0.878, 1.994)	0.243	1.256 (0.856, 1.843)
Antibiotic use timing						
Within 2weeks	0.327	1.228 (0.814, 1.853)	0.194	1.335 (0.863, 2.064)	0.267	1.269 (0.833, 1.934)
Within 1 week	0.328	1.243 (0.804, 1.923)	0.162	1.386 (0.877, 2.19)	0.238	1.296 (0.842, 1.992)
Antibiotic classes						
β-lactam	0.670	1.11 (0.687, 1.796)	0.517	1.183 (0.712, 1.967)	0.696	1.1 (0.68, 1.78)
Other	**0.026***	1.875 (1.08, 3.256)	**0.043***	1.842 (1.019, 3.327)	**0.033***	1.824 (1.051, 3.168)
Antibiotic oral administration	0.345	1.211 (0.814, 1.802)	0.215	1.304 (0.857, 1.985)	0.375	1.197 (0.805, 1.78)
