# Supplementary material for: Impact of Antibiotic Use in the Primary Treatment of Nasopharyngeal Carcinoma
Source: Cancers (Basel). 2026 Jun 26;18(13):2082. doi: 10.3390/cancers18132082 (PMC13359527; doi:10.3390/cancers18132082)
Supplement: Supplementary file 1 [file cancers-18-02082-s001.zip › Supplementary Table S2.pdf]

**Supplementary Table S2 Univariate Cox regression analysis of the factors affecting overall survival status of NPC patients.**

| | **Overall OS** | | **5-year OS** | | **10-year OS** | |
|---|---|---|---|---|---|---|
| | **P value** | **HR, 95% CI** | **P value** | **HR, 95% CI** | **P value** | **HR, 95% CI** |
| Gender | 0.981 | 0.998 (0.822, 1.210) | 0.861 | 0.979 (0.770, 1.244) | 0.924 | 0.991 (0.814, 1.205) |
| Age | **＜0.001*** | 1.042 (1.026, 1.057) | **＜0.001*** | 1.042 (1.023, 1.061) | **＜0.001*** | 1.041 (1.025, 1.057) |
| Smoking | | | | | | |
| Ever smoker | 0.267 | 1.269 (0.833, 1.935) | 0.553 | 1.183 (0.680, 2.059) | 0.204 | 1.315 (0.861, 2.008) |
| Current smoker | **0.031*** | 1.534 (1.039, 2.263) | **0.003*** | 2.008 (1.269, 3.176) | **0.027*** | 1.56 (1.052, 2.313) |
| Alcohol | 0.966 | 1.008 (0.692, 1.468) | 0.873 | 0.963 (0.604, 1.533) | 0.963 | 0.991 (0.677, 1.45) |
| Stage (vs Stage I) | | | | | | |
| Stage II | 0.883 | 1.072 (0.425, 2.701) | 0.321 | 2.867 (0.359, 22.920) | 0.894 | 1.065 (0.423, 2.683) |
| Stage III | 0.370 | 1.467 (0.634, 3.394) | 0.075 | 6.066 (0.835, 44.075) | 0.422 | 1.41 (0.609, 3.267) |
| Stage IV | **0.004*** | 3.419 (1.470, 7.952) | **0.009*** | 13.873 (1.907, 100.939) | **0.006*** | 3.305 (1.420, 7.691) |
| Stage (Early vs advanced) | **0.003*** | 1.926 (1.241, 2.99) | **＜0.001*** | 3.517 (1.767, 7.002) | **0.001*** | 1.845 (1.187, 2.868) |
| LNM | **0.006*** | 2.374 (1.282, 4.396) | **0.004*** | 17.444 (2.431, 125.195) | **0.008*** | 2.301 (1.242, 4.262) |
| NC | **＜0.001*** | 2.368 (1.671, 3.356) | **＜0.001*** | 3.099 (2.051, 4.684) | **＜0.001*** | 2.336 (1.643, 3.322) |
| CCT | 0.441 | 0.848 (0.557, 1.290) | 0.665 | 1.134 (0.642, 2.003) | 0.368 | 0.824 (0.541, 1.255) |
| Abx | 0.136 | 1.288 (0.924, 1.795) | 0.336 | 1.223 (0.811, 1.843) | 0.169 | 1.266 (0.904, 1.771) |
| Abx around primary Tx | 0.063 | 1.37 (0.983, 1.910) | 0.207 | 1.302 (0.864, 1.963) | 0.081 | 1.349 (0.964, 1.888) |
| Antibiotic use timing | | | | | | |
| Within 2weeks | 0.113 | 1.333 (0.834, 1.903) | 0.224 | 1.311 (0.848, 2.026) | 0.060 | 1.412 (0.986, 2.022) |
| Within 1 week | 0.148 | 1.319 (0.907, 1.920) | 0.202 | 1.347 (0.852, 2.128) | 0.096 | 0.852 (0.706, 1.029) |
| Antibiotic classes | | | | | | |
| β-lactam | 0.760 | 1.069 (0.698, 1.637) | 0.943 | 0.981 (0.574, 1.676) | 0.846 | 1.044 (0.677, 1.609) |
| Other | **0.005*** | 1.966 (1.224, 3.157) | **0.015*** | 2.002 (1.143, 3.506) | **0.004*** | 1.997 (1.243, 3.209) |
| Antibiotic oral administration | 0.054 | 1.39 (0.994, 1.943) | 0.17 | 1.335 (0.884, 2.016) | 0.072 | 1.365 (0.973, 1.917) |
