# Supplementary material for: Impact of Antibiotic Use in the Primary Treatment of Nasopharyngeal Carcinoma
Source: Cancers (Basel). 2026 Jun 26;18(13):2082. doi: 10.3390/cancers18132082 (PMC13359527; doi:10.3390/cancers18132082)
Supplement: Supplementary file 1 [file cancers-18-02082-s001.zip › Supplementary Table S3.pdf]

Supplementary Table S3 Treatment period and physical baseline of NPC patients with or without Abx

Characteristics	Total number (n = 455, %)	Antibiotics Yes (n = 191, 42.0%)	Antibiotics No (n = 264, 58.0%)	P value
**Radiotherapy, RT**				
**RT only**	73 (16.0%)	17 (8.9%)	56 (21.2%)	
Average therapy period (Mean±SD, days)	-	105.35±5.85	100.84±15.26	0.080
**Neoadjuvant Chemotherapy, NC**	104 (22.9%)	54 (28.3%)	50 (18.9%)	
Average therapy period of NC	-	35.09±14.49	45.18±23.55	0.242
Average therapy period of RT	-	49.31±8.95	47.86±3.71	0.239
**Concurrent Chemotherapy, CC**	372 (81.8%)	166 (36.5%)	206 (45.3%)	-
**CC only**	278 (61.1%)	119 (62.3%)	159 (60.2%)	-
Average chemotherapy period	-	32.86±8.99	35.06±7.90	0.092
Average radiotherapy period	-	48.74±5.30	47.48±3.35	0.598
**NC+CC**	101 (22.2%)	54 (28.3%)	47 (17.8%)	-
Average therapy period of NC	-	35.36±14.49	43.04±18.67	0.508
Average therapy period of CC	-	34.11±14.91	31.54±9.35	0.226
Average therapy period of RT	-	49.25±9.02	47.54±2.77	0.165
**ECOG Score**[a]				0.166
0	305 (67.0%)	116 (60.7%)	189 (71.6%)	-
1	98 (21.5%)	47 (24.6%)	51 (19.3%)	-
2	5 (1.1%)	3 (1.6%)	2 (0.8%)	-
3	1 (0.2%)	1 (0.5%)	0 (-)	-
4	1 (0.2%)	0 (-)	1 (0.4%)	-
5	1 (0.2%)	0 (-)	1 (0.4%)	-
**KPS Score**[b]				0.115
100	22 (4.8%)	6 (3.1%)	16 (6.1%)	-
90	39 (8.6%)	17 (8.9%)	22 (8.3%)	-
80	3 (0.6%)	2 (1.0%)	1 (0.4%)	-
70	2 (0.4%)	2 (1.0%)	0 (-)	-

[a] ECOG score: Eastern Cooperative Oncology Group (ECOG) Performance Status, which reflects a patient's level of functioning in terms of their ability to care for themself, daily activity, and physical ability.

[b] KPS score: Karnofsky Performance Scale (KPS), which describes a patient’s functional impairment.
